# Paraneoplastic anti-SRP antibody positive immune-mediated necrotizing myopathy in a young female associated with lymphoma.

**DOI:** 10.1177/22143602251327002

**Published:** 2025-04-23

**Authors:** Johanna Heugenhauser, Franziska Di Pauli, Günther Stockhammer, Andreas Chott, Clemens Feistritzer, Daniel Egle, Benjamin Henninger, Julia Wanschitz

**Affiliations:** 1 Department of Neurology, Medical University of Innsbruck, Innsbruck, Austria; 2 Institute of Pathology, Klinik Ottakring, Vienna, Austria; 3 Department of Internal Medicine V, Hematology and Oncology, Medical University of Innsbruck, Innsbruck, Austria; 4 Department of Gynecology, Breast Cancer Center Tirol, Medical University of Innsbruck, Innsbruck, Austria; 5 Department of Radiology, Medical University of Innsbruck, Innsbruck, Austria

**Keywords:** inflammatory myopathy, anti-SRP antibody, lymphoma, paraneoplastic

## Abstract

We report an exceptional case of immune-mediated necrotizing myopathy (IMNM) associated with anaplastic large cell lymphoma (ALCL). A 26-year-old female patient presented with subacute bilateral proximal muscle weakness and myalgia, highly elevated creatin kinase (CK), and seropositivity for anti-SRP antibodies. Tumor screening by FDG-PET/CT detected an enlarged axillary lymph node with high FDG uptake. Histology of the excised lymph node revealed ALCL, positive for ALK and CD30. Therapy with brentuximab, cyclophosphamide and doxorubicin resulted in complete remission of the lymphoma, additional treatment with oral steroids and repeated cycles of intravenous immunoglobulins led to improvement of muscle weakness and normalization of CK. 18 months after diagnosis the patient remains tumor free with mild to moderate residual axial weakness. A literature review of paraneoplastic anti-SRP antibody positive IMNM identified eleven cases of whom five had a tumor diagnosis within a ±3 years window. The majority of patients had different solid tumors except one with a hematological malignancy. Despite the rare association of anti-SRP antibody positive IMNM and malignancy, early extensive tumor screening was crucial for the management of our patient.

## Background

Idiopathic inflammatory myopathies (IIM) are a heterogeneous group of rare autoimmune disorders characterized by muscle weakness and variable extramuscular manifestations.^1,2^ Currently, five different subtypes can be distinguished based on clinical presentation, muscle biopsy findings and/or results of antibody testing.^2^ The occurrence of IIM is associated with increased risk of cancer, with studies reporting a global malignancy rate of 6.7–32%.^1^ Especially solid tumors like colorectal, lung, and ovarian cancers tend to be the leading tumor entity, occurring prior to the diagnosis of IIM,^1^ but the risk for lymphatic and hematopoietic malignancies is also increased as shown by a meta-analysis of cohort studies in dermatomyositis (DM).^3^ Increased tumor-risk is not evenly distributed among IIM subgroups and an association with cancer is mainly observed in patients with DM, in particular in the context of seropositivity for anti-transcriptional intermediary factor 1 gamma (TIF1γ) and anti-nuclear matrix protein 2 (NXP2) antibodies.^1,4^ Immune-mediated necrotizing myopathy (IMNM) has been proposed as another subentity with increased cancer risk,^4^ however this observation was not reproduced by subsequent studies.^5,6^

IMNM has been delineated as a separate entity of IIM by the discovery of two myositis-specific antibodies, anti-signal recognition particles antibodies (anti-SRP) and anti-3-hydroxy-3-methylglutaryl-coA reductase antibodies (anti-HMGCR)^7^ and is characterized by muscle fiber necrosis, regeneration and little or no inflammatory infiltrates on muscle biopsy.^8^ Clinically, patients with IMNM mainly present with symptoms of myalgia and proximal muscle weakness predominantly involving the lower limbs, but little extramuscular immune-mediated damage.^7^ The diagnosis of IMNM can be made on the basis of highly increased serum creatinine kinase (CK) levels, characteristic symptoms and positivity for anti-HMGCR or anti-SRP antibodies,^9^ while in patients without myositis-specific antibodies (approximately 20% of patients) a confirmatory muscle biopsy is mandatory.^7^ According to the recent evidence- and consensus-based recommendations for IIM-associated cancer screening, anti-SRP antibody positivity is rated as a low-risk factor warranting only a basic screening without special imaging.^10^

We report an exceptional case of paraneoplastic anti-SRP antibody positive IMNM in a young patient associated with anaplastic large cell lymphoma (ALCL), a rare non-Hodgkin lymphoma variant of T-cell origin and undertake a literature review of this rare condition.

## Material and methods

### Ethics

Written informed consent was obtained from the patient. The case study was approved by the Ethics Committee of the Medical University of Innsbruck (EK No: 1002/2023)

### Literature review

We conducted a literature search in MEDLINE. The search was restricted to the English language regardless of the publication date. Search terms were: necrotizing myopathy AND paraneoplastic or necrotizing myopathy AND cancer or immune-mediated necrotizing myopathy AND paraneoplastic or immune-mediated necrotizing myopathy AND cancer. In addition, articles identified in reference lists of the individual papers were selected if considered appropriate. We included studies that fulfilled the following criteria: cases of anti-SRP antibody positive IMNM with an additional tumor diagnosis. Cases were then stratified as likely or unlikely tumor associated with respect to occurrence of malignancy within 3 years before or after IIM diagnosis.^10^ Seronegative – or anti-HMGCR antibody positive IMNM cases were not considered.

## Results

### Case

A 26-year-old woman was initially referred in March 2022 to the rheumatology outpatient clinic because of subacute muscle weakness and myalgia lasting for over two months. Her major complains were difficulties in standing up from sitting positions and painful arm elevation with muscle weakness grade 4/5 according to the Medical Research Council scale. Apart from obesity (BMI 35.3), her previous medical history was inconspicuous, and she took no medication. Initial laboratory results showed a strongly elevated creatin kinase (CK) of 8210 U/l (normal values: 26-170 U/l), as well as elevated lactate-dehydrogenase (LDH), aldolase, troponin T (1713.0 ng/l, normal values 0.0–14.0 ng/l) and myoglobin. Troponin I, NT-pro-BNP, electrolytes and C-reactive protein (CRP) were normal. Additional rheumatologic workup revealed strongly positive SRP-antibodies (detected by EUROIMMUN-Immunoblot) and a low ANA titer. At neurological examination the patient presented with symmetrical proximal (lower > upper) limb and axial weakness without facial/bulbar or respiratory involvement. She had no visible muscle atrophy or scapular winging. Electromyography (EMG) demonstrated myopathic muscle action potentials in deltoid and vastus lateralis muscles. Based on the typical clinical presentation and laboratory findings anti-SRP antibody positive IMNM was diagnosed and therapy with oral prednisolone (100 mg/d) was initiated immediately. Computerized tomography (CT) of the chest, echocardiography and magnet resonance imaging (MRI) of the heart showed no evidence of pulmonary or cardiac involvement. However, an enlarged lymph node in the right axilla was described on CT and MRI.

**Figure 1.**
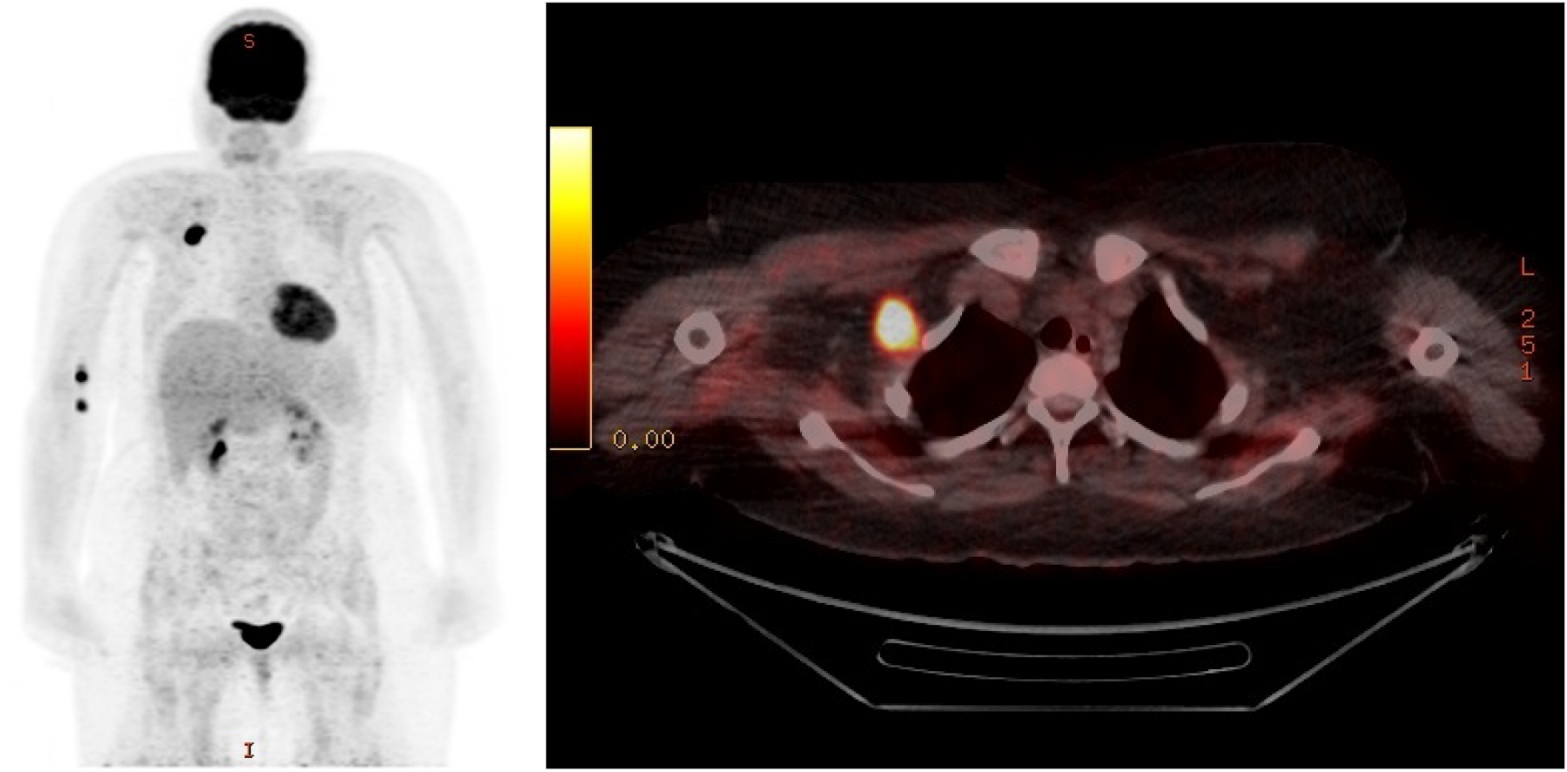
^18^F­FDG-PET/CT image showing a high glucose uptake in the right axillary lymph node, while no other hypermetabolic lesions were detectable (permission was granted by the patient).

**Figure 2.**
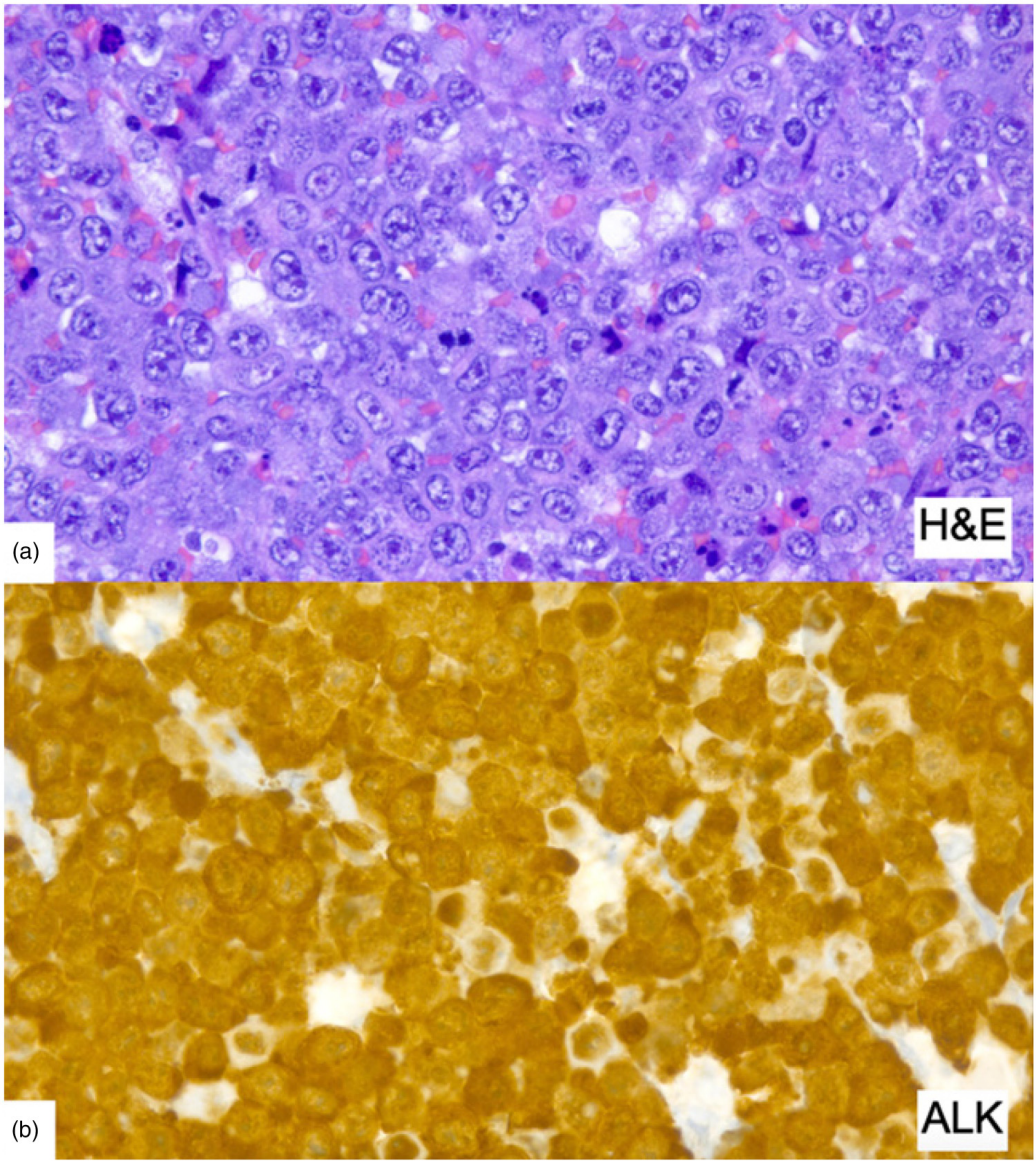
Histopathological findings in the axillary lymph node. (a) Densely packed atypical large lymphoid cells. (b) ALK staining revealed strong cytoplasmic and nuclear reactivity of the large cells.

On follow-up two weeks after the initiation of oral corticosteroid treatment, the patient presented with further increasing proximal muscle weakness of the upper and lower extremities and a persistently elevated CK of 5764 U/l. Intravenous immunoglobulins 2 g/kg per body weight for five consecutive days were added as a fast acting and usually well-tolerated second line treatment. Serologic testing for viral infections e.g., hepatitis B and C, human immunodeficiency virus (HIV), Epstein-Barr virus (EBV) and Cytomegalovirus (CMV) were negative. A breast ultrasound remained inconspicuous for neoplasia. In the 2­^18^F­fluorodeoxyglucose (^18^F­FDG) positron emission tomography (PET) imaging, the right axillary lymph node showed increased ^18^F­FDG tracer uptake, while no other glycose hypermetabolic lesions were seen (Figure 1). A complete resection of the lymph node was carried out with the result of an anaplastic large-cell lymphoma, ALK+, CD30+ (Figure 2). The patient was transferred to the hemato-oncology department and immunochemotherapy with a total of six cycles of Brentuximab, cyclophosphamide and doxorubicin (Brentuximab + CHP) was administered. Oral steroids were maintained during immunochemotherapy at a dose of 100 mg/d and then gradually tapered to 5 mg/d.

**Figure 3.**
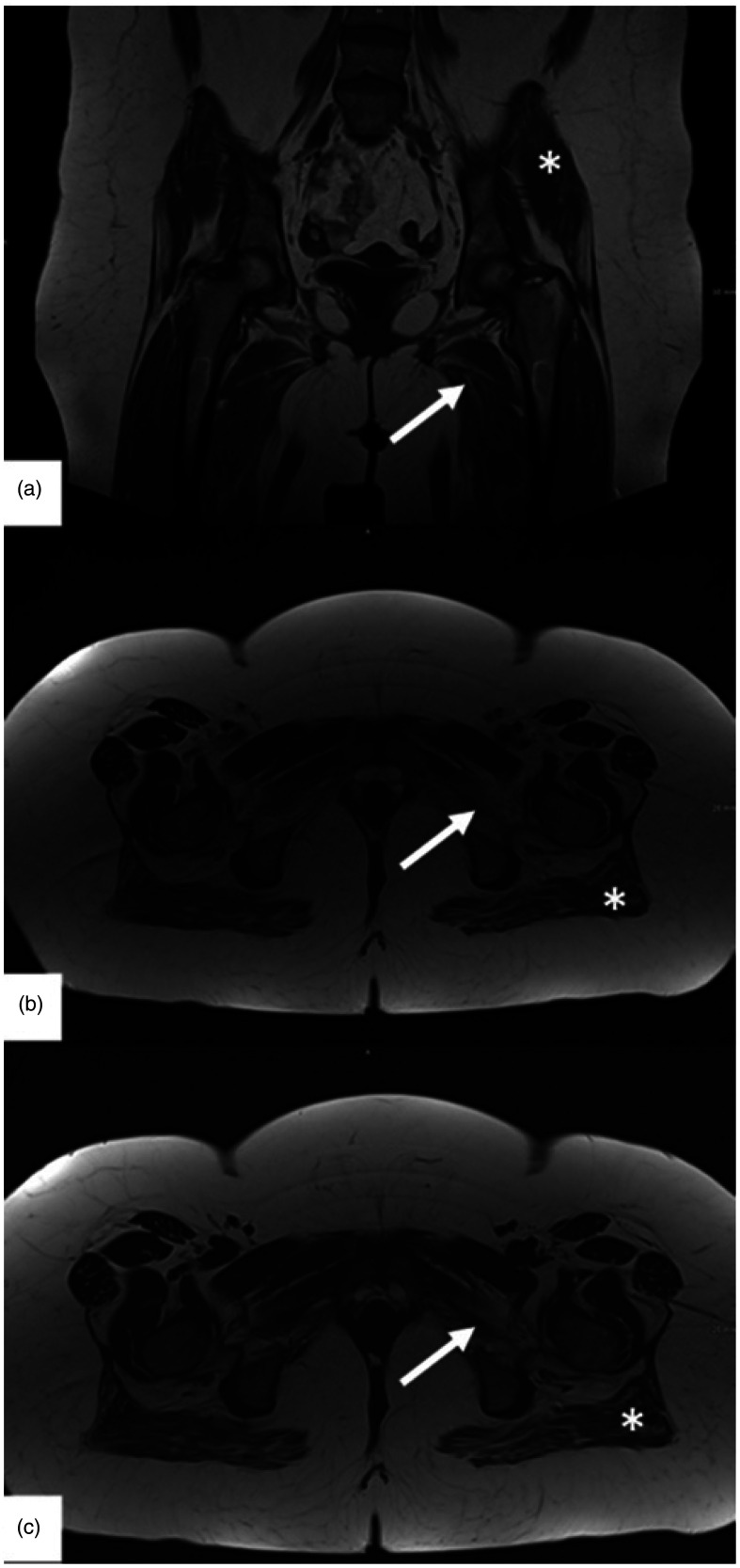
Bilateral fatty atrophy of the adductor muscles (arrow) and the gluteus maximus (*). Edema in the gluteus maximus and adductor muscles. (a) Coronal T1-wheighted image. (b) Axial T2 TSE DIXON in in-Phase. (c) Axial T2 TSE DIXON in Fat only image (permission was granted by the patient).

On follow-up 6 months later, the patient achieved complete hematologic remission, but proximal muscle weakness (MRC 3–4/5) was still persistent, although CK dropped to 292 U/l. Anti-SRP antibodies remained positive at lower intensity on Euroimmune line blot. EMG showed unchanged myogenic damage with minor denervation in the vastus lateralis. MRI of the pelvis and thigh showed muscle edema compatible with myositis and a moderate muscular atrophy as well as minor fatty degeneration (Figure 3). In the health assessment questionnaire (HAQ) the patient reached 14 of 24 points corresponding to a functional disability index (FDI) of 1.75 (range 0–3 points). At this time point, chemotherapy was completed and treatment with two cycles of intravenous immunoglobulins 2 g/kg bodyweight for five days each was readministered, followed by a one-month rehabilitation therapy. At repeated follow-up's at months 9, 12 and 18 the patient presented with mild permanent axial muscle damage (MRC grade 5-/5), normal CK and improved patient reported outcomes (HAQ: 8 of 24 points; FDI: 1 of 3) which remained unchanged after discontinuation of steroids due to weight gain and re-application of 3 cycles of IVIg 1 g/kg per body weight at a 4 weeks interval from October to December 2023. SRP antibodies were retested in another laboratory with a weak positive result. As no additional benefit was achieved by the last 3 IVIg cycles, the patient is currently on weekly physical treatment without specific medication and regular follow-up at the hematology department.

### Literature review

We identified 11 cases of anti-SRP antibody positive IMNM in the literature who had a history of malignant disease.^4,5,11,12^ There were six female and five male patients. The mean age at cancer diagnosis was 56 years. Only one patient had a hematologic malignancy. Two others manifested with breast cancer and the other eight patients were all diagnosed with different solid tumors as summarized and described in more detail in Table 1. In five patients, concomitant cancer occurred within a ±3 years window of IIM diagnosis, while in the remaining six patients a longer time interval up to 13.5 years raises doubt about a paraneoplastic etiology of IIM in these cases.

**Table 1.** Published case reports with the diagnosis of anti-SPR antibody positive immune-mediated necrotizing myopathy (IMNM) and different cancer entities. Only 5 patients had malignancies within ±3 years. IMNM: immune-mediated necrotizing myopathy, AB: antibodies, CK: creatin kinase, Ref.: references, anti-SRP: anti-signal recognition particles antibodies, U/l: Unit/liters, NA: not available.

Gender	Age	Cancer type	IMNM specific AB	Cancer before IMNM diagnosis	Time between cancer and myopathy (years)	Interval between cancer and myopathy	Outcome	CK levels	Muscle biopsy	Cancer therapy	IMNM therapy	Ref.
Male	63	Lung	Anti-SRP	no	0	<3 years	dead	12949 U/l	yes	chemotherapy	prednisone	^11^
Male	79	Hepatocellular	Anti-SRP	yes	1.7	<3 years	death	NA	yes	NA	NA	^4^
Female	73	Breast	Anti-SRP	no	−1	<3 years	alive	NA	yes	NA	NA	^4^
Male	43	Lymphoma	Anti-SRP	yes	3	<3 years	alive	NA	yes	NA	NA	^5^
Female	29	Thymoma	Anti-SRP	no	1.5	<3 years	alive	NA	yes	NA	NA	^5^
Male	67	Bladder	Anti-SRP	no	−3.9	>3 years	alive	NA	yes	NA	NA	^4^
Female	83	Breast	Anti-SRP	yes	3.9	>3 years	alive	NA	yes	NA	NA	^5^
Female	34	Colon	Anti-SRP	yes	7	>3 years	death	NA	yes	NA	NA	^5^
Female	39	Melanoma	Anti-SRP	yes	3.8	>3 years	death	NA	yes	NA	NA	^5^
Male	53	Colon	Anti-SRP	yes	12	>3 years	alive	4846 U/l	yes	surgery	prednisone, immunoglobulins	^12^
Female	55	Endometrial	Anti-SRP	yes	13.8	>3 years	alive	NA	yes	NA	NA	^5^

## Discussion

Whereas an association of cancer and IMNM was mainly reported for seronegative and anti-HMGCR antibody positive patients in a large French cohort,^4^ only a small number of cases of anti-SRP antibody positive IMNM and co-occurrence of malignancy has been published so far^4,5,11,12^ (Table 1). A recent longitudinal referral case-control study analyzing 152 serologically characterized IMNM patients, however, did not observe greater cancer risk in IMNM vs matched controls and cancer rates were not significantly different between serological subtypes, thus contradicting previous findings although a referral bias to a tertiary center might have influenced the results of the study.^5^ In another single-center, retrospective cohort study of 203 patients with a cancer history and IIM, again patients with IMNM had no increased cancer risk compared to the general population.^6^

In general, an age-risk appropriate malignancy screening is recommended for idiopathic inflammatory myopathies.^5,13^ Following recent international guidelines, extensive tumor screening should be performed in the presence of “high risk” factors such as dermatomyositis, age at onset of IIM > 40 years, features of persistent high disease activity despite immunosuppressive therapy, dysphagia, cutaneous necrosis or ulceration, rapid disease onset, positive anti-TIF1γ or anti-NXP2 antibodies, and in patients negative for known myositis-specific autoantibodies.^10,14^ An ^18^F-FDG-PET/CT is preferred, as it is the most sensitive screening approach,^5^ but CT-scanning of the pelvis, thorax and abdomen is also a possibility.^14^ Positivity for anti-SRP antibodies on the other hand is rated as a “low risk factor” and for these cases only a “basic cancer screening” comprising clinical history, physical examination, laboratory tests and a plain chest X-ray radiograph is recommended.^10^ The time of tumor diagnosis is another important factor as a causal relationship with IIM is most likely within three years before or after onset of symptoms.^4,10^ Among the reported cases of IMNM with SRP antibodies from the literature (Table 1), only five out of eleven patients had a diagnosis of cancer and IMNM within a ±3 year window,^4,5,12^ while a longer time interval in the remaining six cases makes a paraneoplastic etiology uncertain. However, it was not possible to identify additional predisposing factors for cancer in those patients, e.g., cytotoxic drugs or other toxic exposures, due to the limited information given. Despite this very rare association of anti-SRP antibody positive IMNM and malignancy^4,7,12^ and absence of the above mentioned risk factors in our patient a FDG-PET/CT was performed for the evaluation of lymphadenopathy shortly after disease onset and facilitated the early diagnosis of concomitant ALCL. This specific type of non-Hodgkin lymphoma is a very rare entity comprising only 15% of all T cell lymphoma diagnosed globally.^15^ There are no studies with a large patient cohort with paraneoplastic syndromes in ACLC and only one case report in the context with polymyositis^16^ has been published so far according to our literature search. Another remarkable feature in our patient was her young age, when the diagnosis of paraneoplastic IMNM was made. With the age of 26 years at onset, she was the youngest patient in comparison to our literature review, and only another patient presented under the age of 30,^17^ whereas the mean age of patients with a paraneoplastic anti-SRP antibody positive IMNM was 57 years (Table 1). According to the literature, idiopathic seropositive IMNM is also mainly diagnosed in patients between 40 and 50 years of age. However, when focusing on the predisposing age for ALK + ALCL, this tumor entity is mainly diagnosed in patients around the age of 30.^15^

Despite the unique characteristics pointed out above, our patient showed very characteristic symptoms for IMNM with the major complains being proximal bilateral muscle weakness and myalgia with a subacute onset over two months. Moreover, very high CK levels (6000–8000 IU/l) are typical for seropositive IMNM and were also seen in our patient. Although, IMNM is described to be mainly a muscle-prominent autoimmune disease, extramuscular immune-mediated damage like interstitial lung disease or myocarditis is seen in up to 40% of patients with positive anti-SRP antibody IMNM,^7^ which was not detectable in our patient clinically as well as on lung CT, cardiac MRI and echocardiography. Due to the typical clinical presentation of IMNM^2,7,9^ and the pattern of muscle edema on MRI of proximal leg muscles a muscle biopsy was not considered necessary for the diagnosis.

Until today, no treatment guidelines are existing for paraneoplastic IMNM. According to consensus guidelines for idiopathic inflammatory myopathies,^14^ the initial therapy should comprise corticosteroids orally or intravenously, based on the severity of symptoms. Since symptoms of most IMNM patients are not well controlled with corticosteroid monotherapy, a second-line therapy with either methotrexate, rituximab or intravenous immunoglobulins should be initiated in those cases.^7,14^ In contrast, patients with paraneoplastic syndromes mediated by surface neuronal autoantibodies usually respond, if immunotherapy and additional oncological treatments are initiated immediately after diagnosis.^18^ Hence, treatment of paraneoplastic IMNM is primarily directed against the specific tumor entity. Moreover, symptom improvement with solely cytotoxic treatment directed against the lymphoma was described in a study of patients with DM or PM before^19^ and in a case of SCLC associated IMNM.^20^ In our patient, marked improvement of muscle weakness and normalization of CK levels was achieved after treatment with corticosteroids, two cycles of intravenous immunoglobulins and six cycles of immuno-chemotherapy. This included Brentuximab, a monoclonal antibody directed against CD30, that is highly expressed on ALCL cells.^21^ As a member of the TNF/nerve growth factor receptor superfamily it is also expressed on activated human CD4+ T-cell clones and other inflammatory cells.^22^ Presence of CD30+ inflammatory infiltrates in IIM muscle biopsies has not been reported so far, however elevated circulating soluble CD30 levels have been detected in early studies in patients with polymyositis and dermatomyositis,^22^ but the precise pathogenic role of sCD30 in IIM remains to be explored. Hence, an ani-inflammatory effect of Brentuximab is unknown , but it might be speculated that it exerted immuno-modulatory activity along with cyclophosphamide, a well-established treatment option for severe IIM.^14^ The good clinical response of our patient is worth mentioning, since seropositive IMNM generally shows a restrained response to therapy, as the disease course is mostly severe and only 50% of patients positive for anti-SRP antibody reach a near-full or full muscle strength after four years of immunotherapy.^7^

In conclusion, our case illustrates an exceptional occurrence of paraneoplastic IMNM with positive anti-SRP antibodies associated with ALCL in a young adult. To our knowledge only one previous case of a paraneoplastic anti-SRP antibody positive IMNM occurring 3 years after the diagnosis of hematologic malignancy was published before.^5^ Although our patient did not meet “high risk” factors for malignancy according to recent guidelines for individual tumor risk stratification in IIM patients^10^ lymphadenopathy was a hint to perform an ^18^F-FDG-PET/CT early at onset of disease which enabled tumor detection and optimal treatment early in the disease course. We conclude that this case should stimulate awareness for hematological malignancies in young IMNM patients. Although we cannot derive general recommendations for tumor screening from our case, we are in concordance with the practice to obtain a CT scan of chest, abdomen and pelvis upon IIM diagnosis in all patients,^6^ while age- and sex- specific cancer screening should be maintained according to individual risk and antibody status.^10^
